# Rooting Out Invasive Species: Lessons from Down Under

**DOI:** 10.1289/ehp.115-a352

**Published:** 2007-07

**Authors:** Tim Lougheed

In a world where heightened airport security is the norm, Australia and New Zealand might seem like a parallel universe. Dogs patrol airport aisles, sniffing not for illegal drugs, but for wayward fruits and vegetables. While the luggage of outgoing passengers may be X-rayed for weapons, incoming visitors have their suitcases scanned for any kind of biological material. You can be required to surrender your shoes, not because they might contain explosives, but because you neglected to clean them off after hiking in some foreign rural area. The security in question is biosecurity—protection against incoming exotic species—and at these checkpoints, no packet of garden seed is too innocent to be seized.

Both countries have powerful incentives for such stringent enforcement. Although Australia and New Zealand are highly urbanized societies, their economies feature a significant agricultural sector that is exceptionally vulnerable to the ravages of invasive species. More recently, though, many observers are adding the prospect of human health issues to the toll exacted by exotics.

In fact, the gauntlet found at these countries’ international airports is just the most obvious aspect of how seriously local authorities and scientists regard the issue of biosecurity. Government policies and academic institutions regularly adopt a unique focus on the way in which plants and animals cross national boundaries, a perspective that may look downright xenophobic at first glance.

Nevertheless, the experience of Australia and New Zealand can provide a valuable example to parts of the world that treat their boundaries in a more casual fashion, at least from a biological point of view. Accomplishments in these countries can offer important lessons to any country that must confront the combined impact of rising volumes of international trade and shifting climate patterns.

## Worlds Apart

The lands Down Under are unique in having developed in physical isolation from other major land masses after splitting from the supercontinent of Gondwana tens of millions of years ago (estimates of just how long ago vary). Odd creatures such as the duck-billed platypus and the kangaroo serve as poster species for a remarkable evolutionary segregation, testifying to a path of biological development and survival well removed from the world’s beaten paths. Flora and fauna in places like Europe, Asia, and the Americas long ago fought for and won ecological niches on their home turf, but some of those battles are just starting to be waged in the antipodes, with the relatively recent influx of exotic species.

When settlers began to colonize these outposts, they regularly spawned such battles by introducing plants or animals in a bid to make a sometimes strange landscape look more familiar to European eyes. The outcome was often devastating, perhaps never more so than following the release of a handful of rabbits in 1859 by an immigrant who wanted to hunt them on his Australian property as he had enjoyed doing in England. The comparatively mild Australian winters meant the rabbits could breed year-round; within a decade the animals had multiplied by the millions, munching on native plants—which had adapted slow growth patterns over millennia of exposure to Australia’s drought-prone climate—and devastating an already parched topsoil. The result was serious erosion and agricultural damage that continues to this day.

Richard Roush, an American entomologist who is now dean of the University of Melbourne’s Faculty of Land and Food Resources, can offer a long list of plants and animals that newcomers brought to the country to replicate their homelands’ gardens, pastures, and livestock. He suggests that as many as half of Australia’s problem species have arrived in this fashion, and that the trend has not waned until comparatively recently.

In research initiated in the early 1990s and published in the September 1994 issue of *Austral Ecology*, the Commonwealth Scientific and Industrial Research Organisation (the country’s leading research body) surveyed instances of some 463 exotic grasses and legumes being deliberately planted in the country’s north between 1947 and 1985. Of that total, 60 species (13%) eventually came to be listed as weeds, including most of the 21 species that also emerged as agriculturally beneficial. While many of the plants surveyed may have held aesthetic appeal for gardeners, only 4 out of the 463 attempts were identified as useful agricultural specimens with no weedy characteristics.

At about the same time this research was being conducted, Roush documented the arrival of the silverleaf whitefly (*Bemisia* spp.) into Australia. He was familiar with this pest’s earlier incursion into California, where it had been transported with shipments of poinsettias. In Australia, Roush called for restrictions on imports of these same plants to forestall the problem, only to be told by Australian authorities that such quarantine efforts could be interpreted elsewhere as a trade barrier under the terms of the General Agreement on Tariffs and Trade. This international treaty sets forth rules for fair and free exchanges of goods between countries. Within a decade, in a March 2003 report titled *Silverleaf Whitefly—Threats and Management Issues for Broad-Acre Crops in Eastern Australia*, Richard Sequeira of the Queensland Department of Primary Industries wrote, “Silverleaf whitefly poses the latest and greatest pest threat to a wide range of agricultural crops in eastern Australia because of its wide host range, high reproduction rate, and its ability to rapidly develop resistance to insecticides.”

“The whole thing was very predictable,” Roush recalls, referring to a profound imbalance between the limited benefit to be gained by individual importers of products that could carry pests and the potentially unlimited costs borne by the society that has to live with those pests afterward. “The asymmetry is that that individual importer doesn’t perceive very much risk, and in the long term he or she is not the one who’s going to have to cover the major costs.”

## Calculating Risks

In Australia, national networks known as Cooperative Research Centres (CRCs) shape the way in which the government assigns resources and expertise to restrict the spread of plants or track animal movements, always with an eye toward heading off trouble before it gets started. By the late 1990s, the CRC for Australian Weed Management had launched a national strategy for dealing with weeds, which later yielded a formal list of 20 “weeds of national significance.”

These 20 plants, taken from a pool of some 3,000 non-native species that have become naturalized within the nation’s borders, were ranked using an assessment system that predicted how quickly and extensively they would spread, and what their impact could be on the environment or the human community. In particular, such weeds are deemed to pose threats to human health, to infrastructure or water supplies, or to agriculture and forest management.

One of the worst offenders is parkinsonia (*Parkinsonia aculeata*), which was introduced as an ornamental shade tree in the early 1900s. Today the tree infests more than 800,000 hectares of land, clogging waterways, blocking livestock access to watering holes, and displacing native vegetation. Another, the ominously named Paterson’s curse (*Echium* spp.), can cause severe allergies and skin irritation in people, as well as overrun pasturelands and poison livestock.

Other invasive species may have even more direct implications for human health. Investigators with the Australian Biosecurity CRC have maintained a military-style vigilance at the narrow Torres Strait that separates the country’s north coast from the islands of Southeast Asia. The focus there is on mosquitoes flying south that could transmit Japanese encephalitis virus (JEV), which has resulted in a handful of cases and two deaths on the Australian mainland since 1995.

The disease does not appear to have become established, but the Australian Quarantine and Inspection Service is taking nothing for granted. Starting in the late 1990s the agency established a colony of sentinel pigs in the region. These animals serve as host to JEV if they are bitten by an infected mosquito.

It could take as long as two weeks to find evidence of the virus in samples of pig blood, however, which is why this expensive program gave way in 2004 to a more efficient system of traps that collect the insects directly, so they can be tested for the JEV antigen. Biosecurity CRC researchers have since taken this strategy a step further, recently developing polymerase chain reaction tools to isolate the antigen from mosquito saliva deposited on a cotton pad in the trap, without the need for testing the insects themselves.

More than a thousand miles to the east across the Tasman Sea, New Zealanders also are battling mosquitoes. Although transmitted ailments such as JEV and dengue fever have not been linked to indigenous mosquitoes in that country, the potential for such an outbreak is being linked to evidence of climate change and the risk of introduction of exotic mosquitoes. Specifically, researchers are weighing the prospect of changes to key factors such as maximum and minimum rainfall thresholds, as well as increasing year-round temperatures, especially the mean mid-winter temperatures that determine how well a species can flourish.

This prospect is already being quantified by members of the International Global Change Institute at the University of Waikato and the Ecology and Health Research Centre at the University of Otago. They have collaborated on an intricate model known as Hotspots, which builds on earlier computer software to estimate how shifting climate patterns could affect New Zealand’s North and South Islands, while adding in new elements to show how those climate patterns would affect the potential distribution of disease-bearing mosquitoes, should they be introduced. The model is described in an April 2005 report titled *Hotspots: Modelling Capacity for Vector-borne Disease Risk Analysis in New Zealand*.

Specific incursions of disease-bearing mosquitoes into New Zealand have been observed over the past decade, although there are still no reported cases of viruses being transmitted in this way. By combining the model with observed data on specific mosquito infestations, these researchers can support the country’s current surveillance methods as well as recommend how those methods should evolve to match the realities of climate change. The results could be crucial to helping local health authorities determine how best to spend their money, and how best to employ their personnel. This information could also serve public health workers on other continents.

## From Grass Roots to the Top of the Food Chain

Beyond merely taking stock of what exotic species might be doing to local agriculture and human health, biosecurity likewise extends to a conscious protection of indigenous plants and animals, with the goal of maintaining the perhaps more stable environment of an earlier time. For example, both countries host a social marketing initiative known as Weedbusters, which is dedicated to raising public awareness of noxious plants and orchestrating efforts to remove them, such as weekend culls by volunteers at sites identified as particularly troublesome.

According to Carolyn Lewis, national coordinator for Weedbusters in New Zealand, the initiative’s greatest service may simply be strengthening the lines of communication and cooperation between various regional bodies, thereby overcoming a tendency to “brand” such efforts at the local level for political purposes. For example, the management committee for New Zealand’s Weedbusters includes representatives from the national Department of Conservation, the Regional Government Biosecurity Managers Group, an NGO called the New Zealand Biosecurity Institute, the Nursery and Garden Industry Association, and the Federated Farmers of New Zealand. “Because we’ve got more than one organization on any one project, you don’t have to be complying with the brand guidelines of each organization,” Lewis says, referring to how seriously some regional agencies take their support and ownership of such efforts.

Educational projects include an “anti-glamour” calendar highlighting some of the most offensive (though often pretty) weeds and a children’s book featuring the Weedbusters mascot, Woody Weed. “Instead of providing a resource for kids in the classrooms,” Lewis explains, “we’ve provided a book that is actually fun enough that kids will read it and get a message. It’s about normalizing that message.”

The message—literally a call to recognize the difference between species that belong and those that do not—has been making its way from the smallest of town councils to the highest levels of scientific and government policy. The response to this message seems to be gaining momentum, with some of the most outstanding victories being claimed on behalf of agriculture. For instance, in the 2006 report *Economic Impact Assessment of Australian Weed Biological Control*, Australian consulting firm AECgroup reviewed 36 biocontrol projects mounted by the CRC for Australian Weed Management, and found that the programs’ total annual budget of AUS$4.3 million was estimated to return almost AUS$100 million in benefits to agricultural producers, their associated communities, and local governments.

Among the most dramatic of those projects was one that built on activities dating back several decades, aimed at controlling ragwort (*Senecio jacobaea*). This introduced species, now found in both Australia and New Zealand, poisons livestock with pyrrolizidine alkaloids, and dairy products from poisoned animals can also harm people. By using biocontrol agents such as the ragwort flea beetle (*Longitarsus jacobaea*), which eats the plant’s roots and is itself an introduced species, losses to ragwort in the hard-hit Australian state of Tasmania were reduced by 84% between 1979 and 1995. According to the AECgroup report, the entire cost of such efforts across the country came to AUS$7.9 million, while the total increase in livestock production in Tasmania alone between 1985 and 2005 came to AUS$19.2 million.

“You won’t find those sorts of returns on the stock market or in real estate,” says Rachel McFadyen, chief executive officer of the CRC for Australian Weed Management. “It is a clear illustration of the results that Australia can expect to obtain from maintaining its national scientific effort and skills.”

## Applying the Lessons Around the World

Much of what has happened in New Zealand and Australia demonstrates the value of putting the implications of invasive species into perspective. Indeed, the first steps in this direction are being taken by North American scientists such as Christina Holzapfel and William Bradshaw, who share a laboratory at the University of Oregon’s Center for Ecology and Evolutionary Biology, where they study mosquitoes collected at dozens of sites between Florida and Canada. Their research, published in the August 2004 issue of *Evolution* and the 9 June 2006 issue of *Science*, explores the evolutionary adaptability of these insects to climate. They are still trying to determine what determines whether a species will thrive, pointing to studies that show a remarkable loss of adaptability in northern mosquitoes that are transplanted into warmer conditions.

At the same time, a workshop held in conjunction with a January 2006 Ecological Society of America conference in Merida, Mexico, issued a call for new and unprecedented levels of cooperation throughout the Americas to monitor the impact of globalization and invasive species. By increasing the volume and compatibility of data being collected locally in various parts of the hemisphere, these participants suggested, information could be shared across borders and potential problems addressed more directly.

The next year, in April 2007, more than 120 U.S. environmental, research, citizen, and sporting groups signed a letter to Congressman Robert W. Bishop urging stronger federal action to prevent the introduction of new invasive species. The letter particularly called for uniform standards for ballast discharges as well as support for screening, control, and educational programs.

Although this work may not reflect a national initiative like those found in Australia or New Zealand, any emerging insights about disease vectors and climate change could be coming none too soon. Just ask Karen Bartlett, an associate professor at the University of British Columbia’s School of Occupational and Environmental Hygiene, who has been studying an airborne fungus that appears to have arid tropical origins in Australia, but has killed eight people in the normally moist, temperate setting of Vancouver Island. The organism was identified as *Cryptococcus gattii* by 2002. It has since been linked with some 165 cases of human illness and 8 deaths going back as far as the late 1990s, and many more deaths and illnesses among wild and domesticated animals. Bartlett described the fungus in the January 2007 issue of *Emerging Infectious Diseases* and the March 2007 issue of *Applied and Environmental Microbiology.*

*C. gattii* is known to be associated with various species of eucalyptus trees, though the nature of the association is not fully understood. On 14 June 2007, a number of U.S. scientists and environmental groups sent a letter to four federal agencies requesting an investigation into the potential human and environmental health risks of genetically engineered cold-tolerant eucalyptus trees being field tested in Alabama. One of the parent species for the genetically engineered hybrids is known to be associated with *C. gattii*.

Because its discovery has been so recent, Bartlett admits that people studying *C. gattii* are still puzzling over how it might have come to Vancouver Island, or whether it has been in the soil for some time, waiting to be released by the warmer, drier summers that have characterized much of the area’s last decade.

What the researchers on the case have learned, she adds, is what the Australians and New Zealanders have long applied in their own work on invasive species—an interdisciplinary approach is the most effective.

“It was a veterinary pathologist who early on knew that there was something different happening, that the disease had a different manifestation,” Bartlett says, noting that this person’s discovery drew in the provincial government body that assembled a comprehensive array of experts. “We put together a team of physicians, veterinarians, and hygienists, and that’s how we got as much information as quickly as we did,” she explains.

And beyond any immediate concerns surrounding *C. gattii*, she adds, the success of this team could point the way toward dealing with a variety of potential threats to human health, regardless of their specific causes.

“That model is the model we’re proposing to be able to very quickly recognize that something is different in the ecosystem,” says Bartlett, who looks upon their current activities as valuable proof of a concept. “We are interested in *Cryptococcus* because of the possibility that it’s going to teach us how to deal with the next invasive alien species—what we would call emerging infectious disease.”

## Cultivating Vigilance

Worldwide, public health investigators could well benefit from what has been learned about the spread of problem organisms in the antipodes. As Roush explains, “Australia and New Zealand have led studies about how to do eradication, or when eradication might still be feasible.”

Perhaps the most significant lesson from any of those studies was published in the January 2007 issue of *Diversity and Distributions* by Dane Panetta, a member of the CRC for Australian Weed Management. He counsels a blend of patience and stubborn determination for anyone engaged in the business of weed eradication, qualities that are bound to prove essential to understanding the real effects not only of exotic invasions, but of climate change generally.

“The evaluation of eradication programs is dependent on information gained through monitoring, and the degree of confidence that can be placed in an evaluation procedure is a function of the reliability of the observations upon which the procedure is based,” he concludes. “Weed eradication programs often require ten years or more to achieve their objective. It is important that progress is evaluated on a regular basis so that programs that are on track can be distinguished from those that are unlikely to succeed.”

## Figures and Tables

**Figure:**
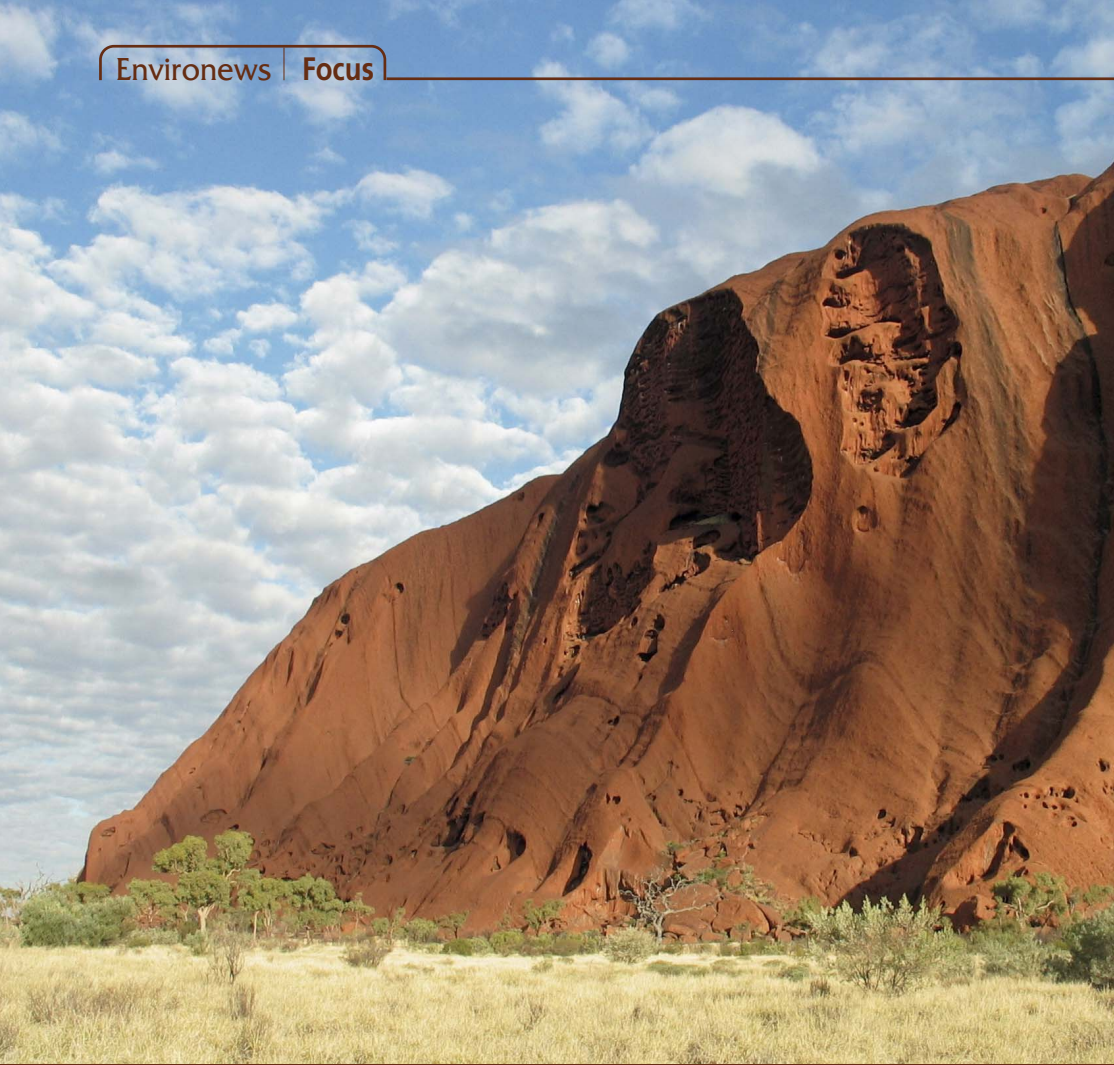
Exotic lands Australia and New Zealand are uniquely vulnerable to the impact of invasive species (such as the gold-colored buffel grass seen encroaching upon Uluru/Ayers Rock, opposite page), and the governments of these countries have made the control of exotic invaders a top priority.

**Figure:**
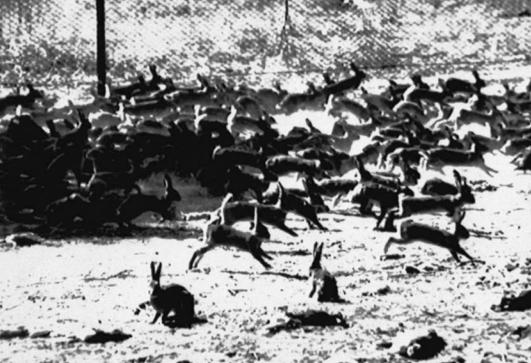
March of the hares In what is perhaps the quintessential cautionary tale about invasive species, millions of rabbits have stripped the Australian landscape of native vegetation since their introduction from Europe in the 1800s.

**Figure:**
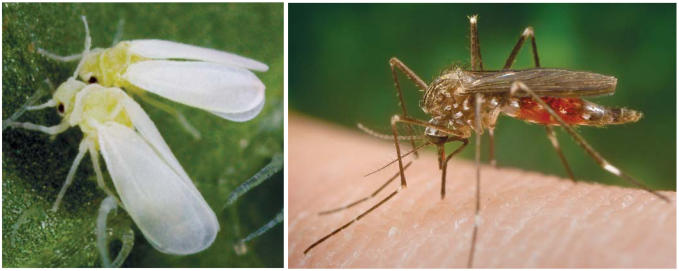
Closing the gates Past introductions of species such as the crop-destroying silverleaf whitefly (left) have been both casual and catastrophic. Today, Australia is watching closely to ensure that mosquitoes carrying Japanese encephalitis virus (right) do not make their way into the country.

**Figure:**
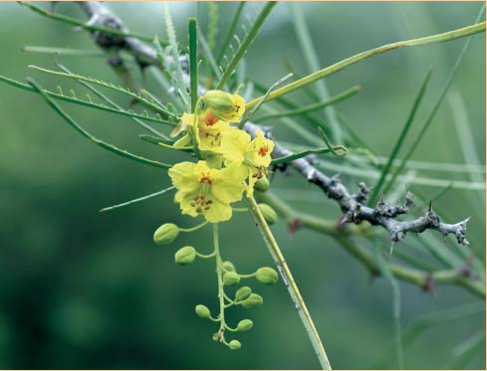
Beauty is skin deep Many plants introduced for their ornamental or shade value, such as parkinsonia, end up taking far more out of the environment than they add.

**Figure:**
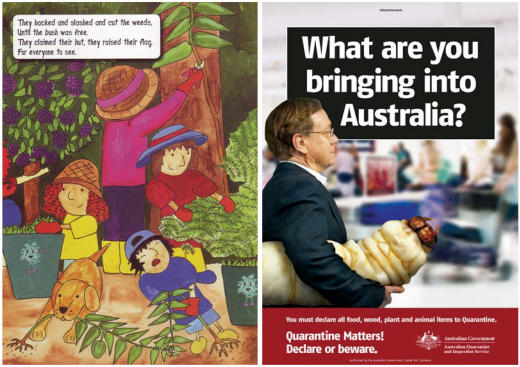
Educational grubstake Public education efforts such as children's books and public service ads convey the message that invasive species are a threat that everyone plays a role in eliminating.

**Figure:**
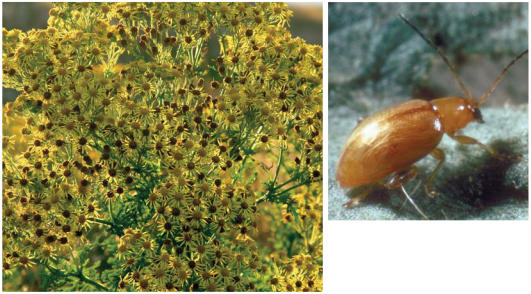
Fighting back Biocontrol agents such as the ragwort flea beetle (which attacks invasive ragwort) are helping reverse the effects of exotic invasions.

